# Use of blinatumomab and CAR T-cell therapy in children with relapsed/refractory leukemia: A case series study

**DOI:** 10.3389/fped.2022.1100404

**Published:** 2023-01-16

**Authors:** Songmi Wang, Aiguo Liu, Na Wang, Yaqin Wang, Ai Zhang, Li Wang, Wen Yu, Chunrui Li, Yicheng Zhang, Qun Hu

**Affiliations:** ^1^ Department of Pediatrics, Tongji Hospital, Tongji Medical College, Huazhong University of Science and Technology, Wuhan, China; ^2^ Department of Hematology, Tongji Hospital, Tongji Medical College, Huazhong University of Science and Technology, Wuhan, China

**Keywords:** acute leukemia, pediatric, immunotherapy, blinatumomab, chimeric antigen receptor T cells

## Abstract

**Background:**

The 5-year event-free survival rate for childhood acute lymphoblastic leukemia (ALL) has increased to more than 85%. However, the 5-year overall survival rate in children with relapsed/refractory ALL did not exceed 50%. In the past decade, immunotherapies (such as blinatumomab and chimeric antigen receptor T-cell therapy) were approved for relapsed/refractory B-ALL, transforming the treatment environment for children with relapsed/refractory ALL.

**Objective:**

This study aimed to explore how immunotherapy can be incorporated into salvage regimens for pediatric patients with relapsed/refractory ALL by retrospectively analyzing the diagnosis and treatment process of seven children with relapsed/refractory leukemia and observing the side effects of the two strategies and long-term survival.

**Methods:**

The clinical features and treatment responses of patients aged <14 years with relapsed/refractory leukemia who received immunotherapy (including Chimeric Antigen Receptor T cell treatment and blinatumomab) at Tongji Hospital, Tongji Medical College, Huazhong University of Science and Technology between February 2014 and April 2022 were retrospectively analyzed.

**Results:**

Seven children underwent immunotherapy. Five patients received immunotherapy and sequential allogeneic hematopoietic stem cell transplantation (HSCT), whereas the other two received only immunotherapy. Five patients achieved complete remission (71.4%). None of the patients had severe cytokine release syndrome. However, one developed grade 3 immune effector cell-associated neurotoxicity syndrome with prior leukoencephalopathy. The median follow-up period was 541 days (range, 186–3,180 days). No deaths were related to treatment. Three patients relapsed, two had CD19-negative recurrences, and the third showed CD19 antigen reduction. One patient died after disease progression, whereas the other died of HSCT-related complications. One patient abandoned the treatment after relapse and was lost to follow-up.

**Conclusion:**

Blinatumomab and CAR T-cell therapy showed excellent remission rates and manageable toxicity in pediatric patients with relapsed/refractory leukemia. However, the duration of the remission was limited. Therefore, further prospective randomized clinical studies should be conducted to improve the long-term efficacy of immunotherapy.

## Introduction

Acute lymphoblastic leukemia (ALL) is the most common malignancy among children. The current risk-directed stratified chemotherapy has improved the 5-year event-free survival (EFS) rate for pediatric patients with ALL by more than 85% (1). However, outcomes in patients with relapsed/refractory ALL remain poor (2, 3). Time from diagnosis to relapse, site of relapse, immunophenotype, and minimal residual disease (MRD) levels with reinduction therapy are all risk factors affecting survival rates after the first relapse (2). Intensive cytotoxic chemotherapy, radiation therapy, and hematopoietic stem cell transplantation (HSCT) are among the conventional treatments for patients with relapsed or refractory diseases. Unfortunately, many children cannot undergo HSCT because of serious adverse events from previous treatment or an inability to achieve deep remission with intensive chemotherapy. In addition, poor outcomes were predicted in patients with pre-transplant positive MRD after HSCT (4). Therefore, improving the induction remission rate of relapsed/refractory ALL is key to achieving favorable outcomes.

Several clinical studies have demonstrated that immunotherapy has a high response rate in patients with relapsed/refractory ALL, which bodes well for pediatric patients with relapsed/refractory ALL. Several promising immunotherapies, such as blinatumomab and Chimeric Antigen Receptor (CAR) T cell therapy, have been approved for relapsed/refractory ALL. Therefore, this study aimed to explore how to incorporate immunotherapy drugs into the rescue regimen of pediatric patients with relapsed/refractory ALL and to investigate the long-term survival and side effects of the two-immunotherapy methods. This was achieved by retrospectively analyzing the clinical features and therapeutic effects of CAR T-cell therapy and blinatumomab in children with relapsed/refractory leukemia.

## Patients and methods

### Patients

Patients were aged <14 years with relapsed/refractory leukemia who received immunotherapy (including CAR T-cell therapy and blinatumomab) at Tongji Hospital, Tongji Medical College, Huazhong University of Science and Technology were included in the study between February 2014 and April 2022. The last follow-up was performed on October 30, 2022.

### CAR T-cell treatment protocol

All patients and their guardians provided signed informed consent before receiving anti-CD19 CAR T-cell or anti-CD19/anti-CD22 CAR T-cell cocktail treatment. Patients receive HSCT depending on the choice of the parents or guardians, disease status, and affordability of the treatment. The Institutional Review Committee of Tongji Hospital, Tongji Medical College, Huazhong University of Science and Technology approved the design of this study (ChiCTR-OPN-16008526, ClinicalTrials.gov#NCT04888468). Clinical investigations were conducted in accordance with the principles of the Declaration of Helsinki. CD19/CD22 CAR with CD28 and 4-1BB costimulatory domains was generated using lentiviral vectors, as previously reported (5). CD19 CAR is used to screen the hinge region by improving the polypeptide (single-chain fragment variable, scFv) that recognizes human CD19 antigens by combining CD137/CD3 intracellular costimulatory signals. All CAR T cells required quality control before discharge.

Anti-CD19/anti-CD22 CAR T-cell cocktail treatment eligibility criteria were as previously reported (5). The target dose for enrolled patients was 2.5 × 10^6^/kg, with each type of CAR T-cell divided into two equal doses. In patients with severe comorbidities or a high disease burden, anti-CD19/anti-CD22 CAR T cells were infused at increasing doses.

The following eligibility criteria were used to determine anti-CD19 CAR T-cell treatment for the included patients (6): 3–21-year-old patients with relapsed/refractory B-ALL; patients with Ph + ALL received at least two TKI treatments; for allogeneic HSCT recipients, the following criteria were met: Allo-HSCT requiring ≥6 months before anti-CD19 CAR T-cell infusion, and no graft-vs.-host disease (GVHD) of grade 2 or above occurred within 2 weeks before PBMC collection; good organ function and survival assessment over 12 weeks; Karnofsky performance status of >60%; and confirmation of the expression of target antigens CD19 on malignant B cells using flow cytometry (FCM) or immunohistochemistry. Patients with uncontrolled infection or definite central nervous system pathology (excluding inactive CNS lesions) were excluded. The study planned to set up three dose groups, adopting a dose-escalating 3 + 3 design (6).

**Figure 1:**
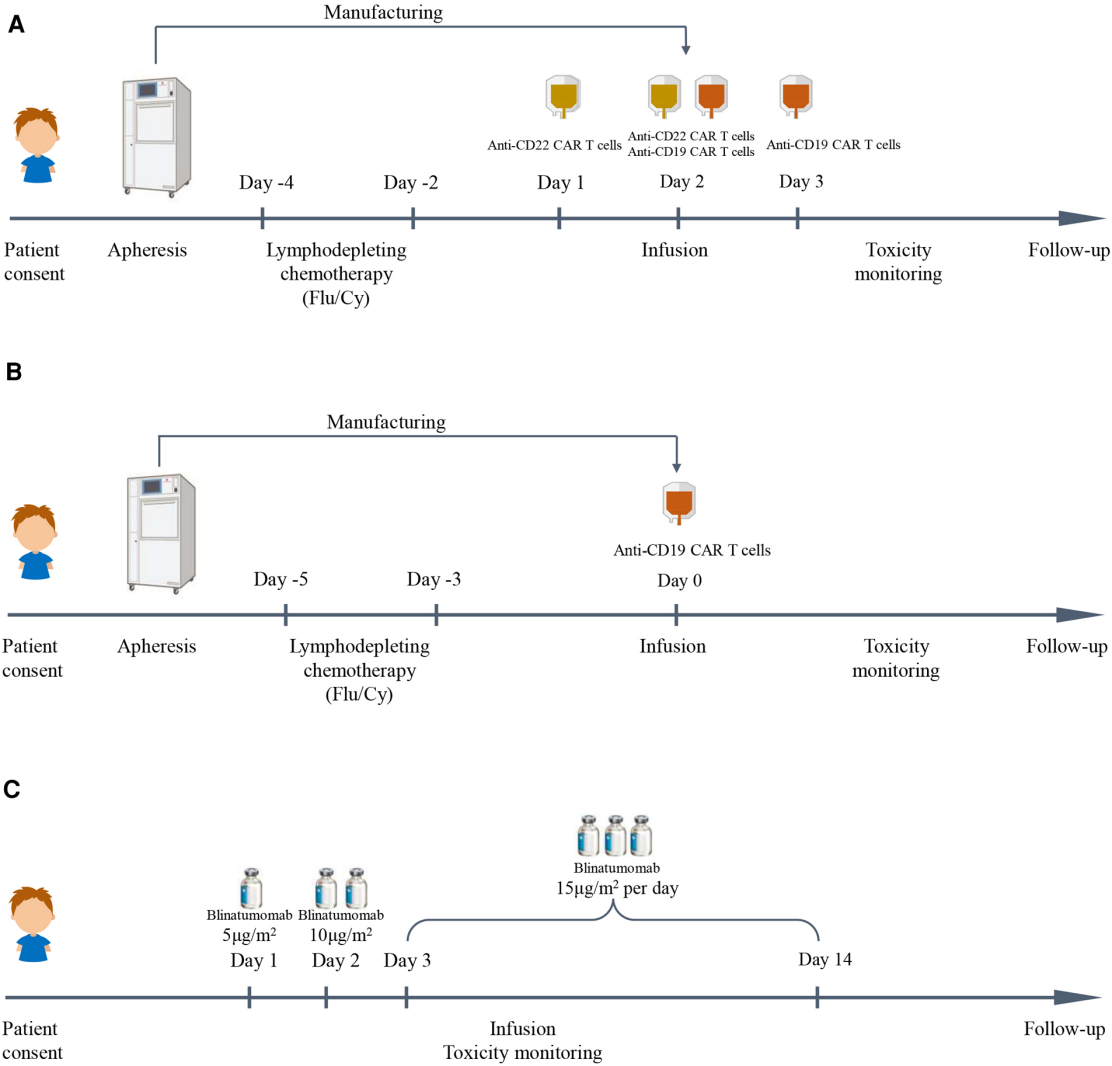
Immunotherapy schedules in this study. (**A**) Anti-CD19/anti-CD22 CAR T cell cocktail treatment schedule. (**B**) Anti-CD19 CAR T cell treatment schedule. (**C**) Blinatumomab treatment schedule.

Patients were administered an FC regimen (fludarabine 25 mg/m^2^/day, cyclophosphamide 20 mg/kg/day) for 3 days (−4 to −2 days or −5 to −3 days) for lymphodepleting chemotherapy. Anti-CD19/anti-CD22 CAR T-cell cocktail treatment was infused with anti-CD19 CAR T cells and anti-CD22 CAR T cells for several consecutive days from day 0. In addition, anti-CD19 CAR T-cell therapy was infused with a single dose of autologous anti-CD19 CAR T cells on day 0. The details are shown in Figure 1.

### Transplant protocol

The patients underwent a myeloablation regimen using BU/CY/ATG (7) for haploidentical HSCT. Individual transplant physicians determine the prophylactic treatment regimen (such as cyclosporine, methotrexate, and mycophenolate mofetil) for GVHD based on disease and transplant-related factors.

### Evaluation of toxicity

The cytokine release syndrome (CRS) grading system was used to grade CRS (8). The Cornell Assessment of Pediatric Delirium is recommended for children aged <12 years to aid in the overall grading of immune effector cell-associated neurotoxicity syndrome (ICANS) (9, 10). Moreover, the Immune Effector Cell-Associated Encephalopathy score is used to assess encephalopathy in adolescents aged >12 years (8).

### Evaluation of response

Morphological analysis and multicolor FCM were used to assess treatment response. Complete remission (CR) was defined as having <5% bone marrow blasts, no circulating blasts, and no extramedullary disease sites. MRD-negative bone marrow blasts accounted for <0.01% of all samples analyzed using multicolor FCM. After CR, the reappearance of blasts in the blood, bone marrow, or extramedullary sites is considered to indicate a relapsed disease.

## Clinical characteristics

**Figure 2:**
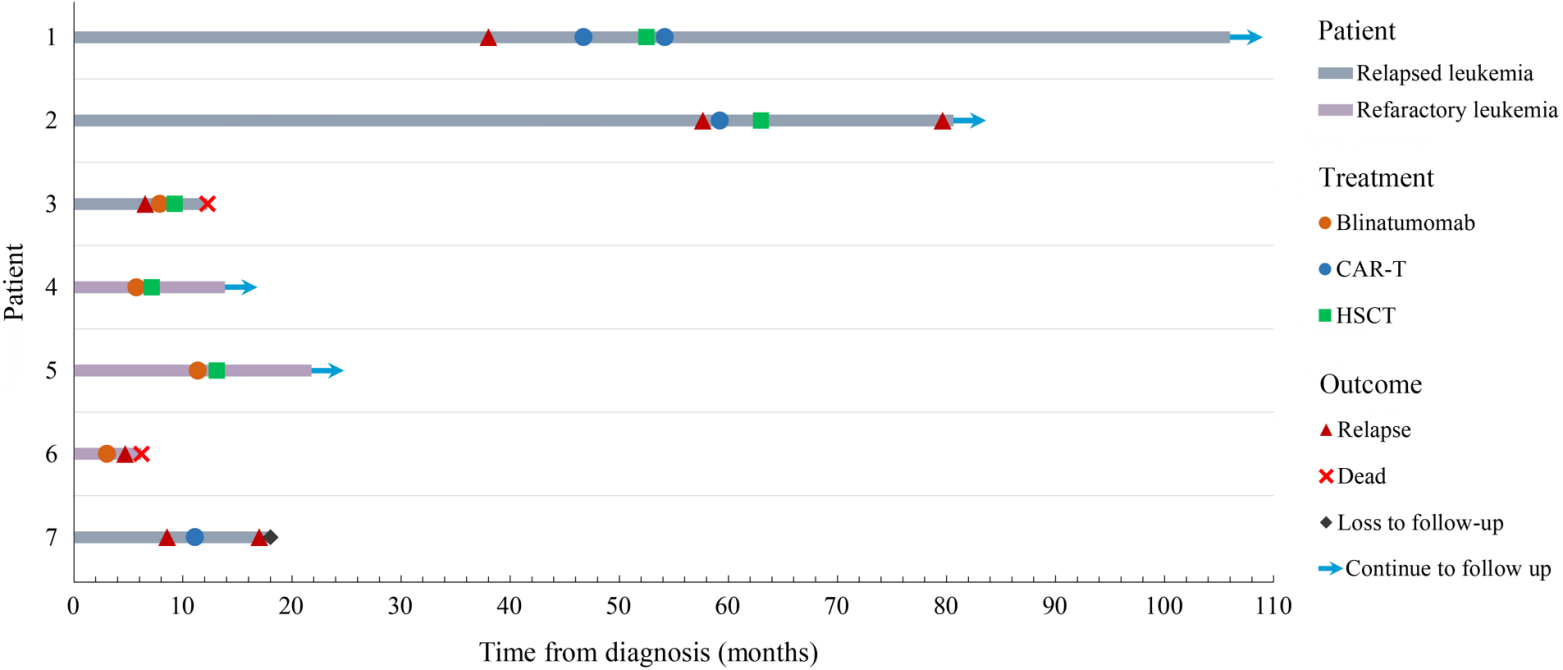
Swimmer plot of patients’ treatment and outcome.

Overall, seven children were enrolled in this study, two (case 6, case 7) of whom received only immunotherapy and five (case 1-case 5) received immunotherapy and HSCT. The detailed clinical characteristics are shown in Tables 1, 2. Five patients achieved complete remission (71.4%). None of the patients had severe CRS. However, one developed grade 3 immune effector cell-associated neurotoxicity syndrome with prior leukoencephalopathy. The response and toxicity related to immunotherapy is shown in Table 3. The median follow-up period was 541 days (range, 186–3,180 days). No deaths were related to treatment. Three patients relapsed. Figure 2 depicts the swimmer plot of patients' treatment and outcome.

**Table 1:** Clinical characteristics of seven patients at baseline.

Patient number	Age/year, sex	Cytogenetics	CNS at diagnosis	Disease status	CNS at relapse
1	5.58, F	Highly complex mixed abnormal karyotype	CNS1	Late relapse	CNS1
2	2.75, M	TEL-AML1 fusion, 11q-	CNS1	Late relapse	CNS3
3	0.33, F	KMT2A rearrangement, KARS mutation	CNS2	Early relapse	CNS1
4	7.92, F	TCF3-ZNF384 fusion	CNS1	Refractory	/
5	4.17, M	BCR-ABL fusion	CNS1	Refractory	/
6	0.4, M	KMT2A rearrangement	CNS2	Refractory	/
7	11.83, M	KMT2A rearrangement, TP53 mutation	CNS1	Early relapse	CNS1

CNS 1, no blasts in cerebrospinal fluid (CSF); CNS 2, <5/μl white blood cells and detectable lymphoblasts in CSF but without clinically evident neurological lesions; CNS 3, ≥5/μl white blood cells and detectable lymphoblasts in CSF or with clinically evident neurological lesions;Early relapse, medullary relapse time ≤36 months from initial B-ALL diagnosis; Late relapse, medullary relapse time >36 months from initial B-ALL diagnosis; /, not applicable.

**Table 2:** Immunotherapy characteristics, HSCT characteristics, and outcomes.

	Immunotherapy characteristics								HSCT characteristics							Outcomse at last follow-up	
Patient number	Days from diagnosis	Type	Pre- blasts, %	Pre-MRD, %	Post-MRD, %	CRS	ICANS	Post infection	Days from Immuno-therapy	neutrophil engraftment	Acute GVHD	Chronic GVHD	Pre-MRD, %	Post-MRD, %	Post infection	Disease status	Survival status
1	1402	Anti-CD19/anti-CD22 CAR T cell cocktail therapy	81	63.2	<0.01	grade 2	No	Yes (pneumonia)	173	10	I Skin	Yes	<0.01	<0.01	CMV, EBV, PC	Complete remission	alive
2	1777	Anti-CD19 CAR T cell therapy	88	72.4	<0.01	grade 2	No	Yes (enteritis)	113	10	I Skin	No	<0.01	<0.01	HHV-5B,HHV-6B, CA	Relapse	alive
3	236	Blinatumomab	0.8	0.01	<0.01	None	No	No	41	12	IV (Skin,Gut)	/	<0.01	<0.01	ADV, CMV, EBV, HHV-6	Complete remission	dead (HSCT-related complications)
4	172	Blinatumomab	0.4	0.01	<0.01	None	No	No	42	10	No	No	<0.01	<0.01	CMV	Complete remission	alive
5	341	Blinatumomab	0.4	2.14*	1.07*	grade 1	Yes (seizures)	No	52	13	No	No	1.07*	<0.01	CMV	Complete remission	alive
6	91	Blinatumomab	5.2	0.65	0.01	grade 1	No	Yes (bronchitis)	/	/	/	/	/	/	/	Relapse	dead
7	333	Anti-CD19 CAR T cell therapy	70	34.3	<0.01	grade 2	No	Yes (URI)	/	/	/	/	/	/	/	Relapse	loss to follow up

ADV, adenovirus; CA, Candida albicans; CMV, cytomegalovirus; CRS, cytokine release syndrome; EBV, Epstein-barr virus; GVHD, graft-versus-host disease; HHV, human herpes virus; ICANS, immune effector cell–associated neurotoxicity syndrome; PC, pneumocystis cainii; URI, upper respiratory tract infection.

* BCR-ABL fusion transcript levels; /, not applicable.

**Table 3:** Response and toxicity of immunotherapy.

Characteristics	Blinatumomab (*n* = 4)	CAR T cell therapy (*n* = 3)	All patients (*n* = 7)
CR	2 (50%)	3 (100%)	5 (71.4%)
CRS	2 (50%)	3 (100%)	5 (71.4%)
Grade1	2 (50%)	0	2(28.6%)
Grade2	0	3 (100%)	3 (42.9%)
Grade ≥ 3	0	0	0
ICANS	1 (25%)	0	1 (14.3%)
Immunotherapy-related infection	1 (25%)	3 (100%)	4(57.1%)
Immunotherapy-related death	0	0	0

CR, complete remission; CRS, cytokine release syndrome; ICANS, immune effector cell–associated neurotoxicity syndrome.

**Figure 3:**
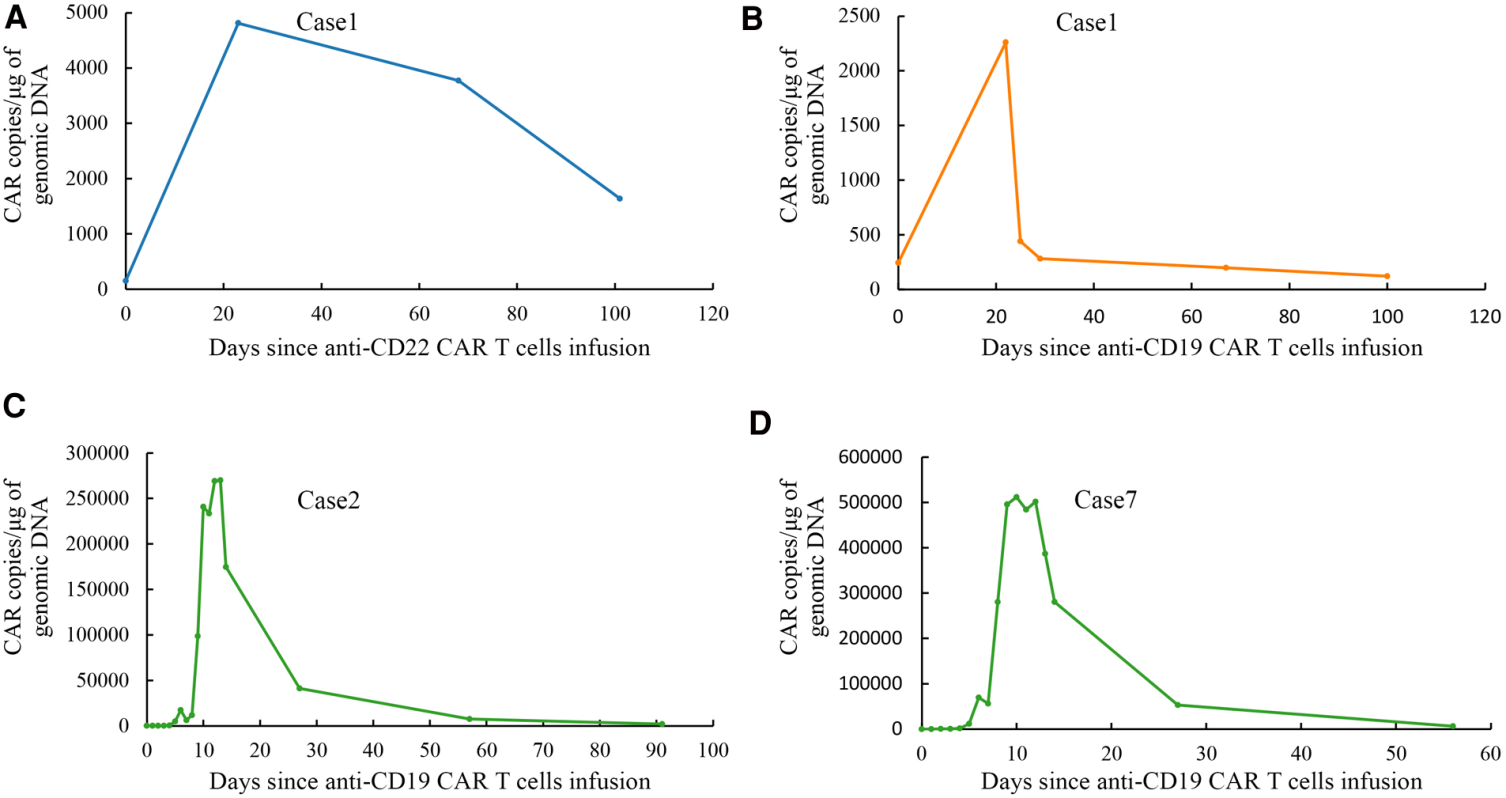
Droplet digital polymerase chain reaction analysis of CAR T cells expansion and persistence *in vivo*. (**A**) Expansion and persistence of anti-CD22 CAR T cells in peripheral blood of case 1. (**B**) Expansion and persistence of anti-CD19 CAR T cells in peripheral blood of case 1. (**C**) Expansion and persistence of anti-CD19 CAR T cells in peripheral blood of case 2. (**D**) Expansion and persistence of anti-CD19 CAR T cells in peripheral blood of case 7.

**Case 1**: A girl aged 5 years and 7 months presented to the hospital with bone pain and decreased platelet count. The patient was diagnosed with ALL (B line, intermediate risk) and subsequently underwent routine chemotherapy in accordance with the CCLG-2008 regimen (11) based on a precise diagnosis of leukemia, which included cell morphology, immunology cytogenetics, and molecular biological typing (MICM). Unfortunately, bone marrow relapse occurred 37 months after diagnosis. After providing informed consent, the patient was enrolled in a clinical trial (ChiCTR-OPN-16008526). The FC regimen was used to remove lymphocyte chemotherapy before infusion, and then the patient received anti-CD19/anti-CD22 CAR T-cell cocktail treatment (day 0, anti-CD22 CAR T cells 1 × 10^6^/kg; day 1, anti-CD22 CAR T cells 1 × 10^6^/kg and anti-CD19 CAR T cells 1 × 10^6^/kg; and day 2, anti-CD19 CAR T cells 1 × 10^6^/kg). The poor expansion of anti-CD19/anti-CD22 CAR T cells in the patient's peripheral blood was shown in Figures 3A,B. Grade 2 CRS occurred on the fourth day after infusion, and the patient responded favorably to treatment with plasmapheresis and glucocorticoids. She attained CR with MRD-negativity 4 weeks later, and the status persisted for 5 months. The child underwent haploidentical donor HSCT (pretreatment protocol: AC/BU/CY/ATG) after 173 days of CAR T-cell treatment because of the poor expansion of CAR T cells. She developed acute chemotherapeutic side effects in the mucosa (toxicity WHO grade 2) and skin (toxicity WHO grade 2). Neutrophil engraftment was achieved 10 days after HSCT. Signs of mild acute GVHD (grade I, skin) appeared on day 24 after the transplant. Lymph node enlargement with Epstein-Barr virus (EBV) activation occurred on day 42 after transplant, and a lymph node biopsy revealed a pathological diagnosis of post-transplant lymphoproliferative disease (polymorphic). She received donor-derived anti-CD19/anti-CD22 CAR T-cell cocktail treatment (day 1, anti-CD22 CAR T cells 1 × 10^6^/kg; day 2, anti-CD19 CAR T cells 1 × 10^6^/kg) after rituximab and immunosuppression reduction were ineffective. Fever was gradually controlled, the lymph nodes were reduced, and the EBV-DNA test result returned negative. Following HSCT, the child developed recurrent upper respiratory tract infection, pneumonia, and infections caused by cytomegalovirus (CMV), EBV, and *Pneumocystis jirovecii*. One year after HSCT, the full classes of immunoglobulins showed that the absence of immunoglobulins (IgG < 0.3 g/L, IgA < 0.07 g/L, IgM < 0.04 g/L) was lower than normal, the proportion of B-lymphocytes was 0.02% (3%–19%), and the full classes of immunoglobulins were still lower than normal (IgG 3.5 g/L, IgA < 0.07 g/L, IgM 0.05 g/L). She continued to achieve clinical remission at the last follow-up.

**Case 2:** A boy aged 2 years and 9 months had a yellowish or pale skin tone and was diagnosed with ALL (B, intermediate-risk, TEL-AML1) according to the leukemia MICM classification. The patient subsequently underwent regular chemotherapy using the CCCG-ALL-2015 regimen (12). Unfortunately, 4.8 years after the initial diagnosis, he experienced a relapse that manifested as bone marrow and central relapse (CNS3). CAR T-cell therapy was a better option for the patient due to its benefits in extramedullary relapsed ALL. After informed consent, he was enrolled in a clinical trial (NCT04888468). The FC regimen was used to remove lymphocyte chemotherapy before infusion; then, he received an infusion of autologous anti-CD19 CAR T cells (2.0 × 10^6^/kg infusion). The expansion of anti-CD19 CAR T cells was validated in the peripheral blood of the boy, as shown in Figure 3C. He developed grade 2 CRS (fever and hypotension) 3 days after infusion, which was relieved after symptomatic treatment with glucocorticoids and rehydration. The patient developed rotavirus enteritis while receiving perioperative CAR T-cell therapy. Four weeks later, he achieved CR with MRD-negativity, which lasted 4 months after infusion. He underwent haploidentical HSCT (pretreatment regimen: VP/BU/CY/ATG regimen) 131 days later. He developed only mild acute GVHD (grade I, skin) after HSCT with no other serious complications. After HSCT, he developed multiple upper respiratory tracts and lung infections caused by the human herpes virus HHV-5B and HHV-6B and *Candida albicans*. One year after HSCT, it was observed that complete classes of immunoglobulin showed that only IgA level was lower than normal (<0.07 g/L). Analysis of lymphocyte subsets revealed that the proportion of B-lymphocytes increased slightly by 21.03% (normal range 3%–19.0%). Unfortunately, the patient experienced a bone marrow relapse approximately 499 days after HCST.

**Case 3:** A 4-month-old girl with skin ecchymosis was brought to our hospital. She was diagnosed with ALL (B, high-risk, CNS2, MLL-ENL) based on the leukemia MICM classification and was being routinely treated in accordance with the CCCG-ALL-2020 protocol. Unfortunately, the use of consolidation resulted in bone marrow relapse. Subsequently, after receiving mitoxantrone reinduction therapy, she achieved morphological CR with persistently positive MRD. We recommended blinatumomab with bridging transplantation as an ideal treatment due to the challenges in producing CAR T cells in infants and the safety of blinatumomab. Blinatumomab (day 1, 5 µg/m^2^; day 2, 10 µg/m^2^; and days 3 and 14, 15 µg/m^2^) was used to treat her for 2 weeks without obvious CRS and ICANS, and MRD. However, she turned negative approximately 2 weeks after the first infusion. The child underwent haploidentical HSCT (pretreatment protocol: VP/BU/CY/ATG) 41 days later. During perioperative HSCT, the child experienced mucositis and infectious diarrhea. The pathogens involved were HHV6 and adenovirus infections. Neutrophil engraftment was achieved 12 days after HSCT. Approximately 3 weeks after HSCT, the patient achieved CR with MRD-negativity. However, the child developed CMV and EBV activation and had a variety of complications, including GVHD grade IV (skin, intestine), transplant-related thrombotic microangiopathy, severe pneumonia, respiratory distress syndrome, hemorrhagic cystitis, and gastrointestinal bleeding. The patient died of transplant-related complications 90 days after transplantation.

**Case 4:** A girl aged 7 years and 11 months with nasal bleeding and ecchymosis of both lower limbs was diagnosed with ALL (B series, intermediate risk) according to the leukemia MICM classification. Subsequently, the patient underwent CCCG-ALL-2020 chemotherapy. After consolidation therapy, her disease was considered refractory to ALL due to a positive MRD of 0.01%. Her family chose blinatumomab for financial reasons. She received blinatumomab treatment (day 1, 5 µg/m^2^; day 2, 10 µg/m^2^; and days 3–14, 15 µg/m^2^) for 2 weeks without obvious signs of CRS and ICANS. After treatment, the patient achieved CR with a negative MRD. She subsequently underwent haploidentical HSCT, during which the child was thought to develop an ATG-related fever and rash that were considered. Neutrophil engraftment was achieved 10 days after HSCT. The child experienced grade 1 mucositis and suffered from CMV activation and hemorrhagic cystitis. Approximately 3 weeks after HSCT, the patient achieved CR with MRD-negativity. Furthermore, the patient was still alive with no adverse events at the last follow-up.

**Case 5**: A boy aged 4 years and 2 months presented to our hospital with a fever. The patient was diagnosed with ALL (B series, intermediate-risk, BCR-ABL fusion) according to the leukemia MICM classification. He subsequently underwent dasatinib treatment and chemotherapy with CCCG-ALL-2020, and the latter caused him to experience leukoencephalopathy. The patient was diagnosed with refractory ALL after a continuous positive quantitative analysis of the BCR/ABL gene and an upward trend (from 0.5% to 2.14%) in bone marrow re-examination. We recommend blinatumomab for inducing treatment because it has fewer adverse events than that in CAR T-cell therapy. Blinatumomab treatment (day 1, 5 µg/m^2^; days 2–3, 10 µg/m^2^; and day 4, 15 µg/m^2^) was a bridging therapy before HSCT treatment. On the fourth day of treatment, the patient experienced seizures lasting for 1 min. Symptoms were controlled following drug discontinuation and antiepileptic treatment. This was considered ICANS. The expression of the fusion gene BCR-ABL P190 was 1.07% following blinatumomab treatment. The child underwent haploidentical HSCT (pre-protocol VP/Bu/Cy/ATG), and neutrophil engraftment was achieved 13 days following HSCT. Approximately 3 weeks after HSCT, the patient achieved CR with MRD-negativity. He experienced CMV activation and hemorrhagic cystitis (grade 3) 43 days after HSCT, and there were no other severe complications, such as GVHD. Blood was observed in the stool of the child due to improper diet 6 months after HSCT, which was followed by GVHD and TMA, which improved after anti-inflammatory and plasmapheresis treatment. The patient was alive with no adverse events until the last follow-up.

**Case 6:** A 3-month-old male child presented to the hospital with a skin mass and a fever. One week after birth, he was diagnosed with myeloid sarcoma by biopsy of a skin mass, and immunohistochemical staining showed the following tumor cells: CD33 (+), CD15 (+), lysozyme (partial +), CD68 (+), CD123 (+), CD99 (+), CD117 (+), ERG (+), CD43 (+), CD34 (−), CD13 (−), TdT (−), MPO (−), CD3 (−), CD5 (−), CD7 (−), Ki67 (LI70%). However, the patient received no further treatment. The bone marrow was rich, infiltrated by 95% blast cells and 1% POX staining, and it was PAS-positive. Using FCM, it showed two distinct groups, with approximately 36.2% of cells (accounting for all nucleated cells) classified as abnormal naive lymphocytes, expressing some B-lineage markers (CD19+, cCD79 dim, CD38+, CD123+, CD9part+, CD24part+) and approximately 54.5% of cells (accounting for all nucleated cells) classified as abnormal myeloid primitives that express some markers (CD38, CD64, CD33, CD123dim, CD117, CD11b, CD15, CD13dim), combined with MICM typing are used to diagnose mixed-phenotype acute leukemia(MPAL) (B/M, MLL-AF10, CNS2). The patient could not achieve CR after receiving multiple lines of chemotherapy. The blast was found in 5.2% of the bone marrow, while MRD was indicated to be approximately 0.65% of abnormal naive B-lymphocytes expressed CD19, CD38, CD123dim, and CD81dim but did not express CD10, CD20, CD34, CD13, CD33, and CD117. The MLL/AF10 quantification was 1.57%. CAR T-cell manufacturing is difficult and time-consuming; therefore, we recommended that blinatumomab induces deep remission before HSCT. Therefore, he received blinatumomab treatment 4 weeks (day 1, 5 µg/m^2^; day 2, 10 µg/m^2^; and days 3–28, 15 µg/m^2^). Grade 1 CRS (fever) started on the second day of treatment using blinatumomab and this was controlled by anti-inflammatory therapy with methylprednisolone. Assessment of bone marrow morphology revealed a severely suppressed myeloid image with an MRD of 0.01% after treatment with blinatumomab. Medium-dose cytarabine (2 g/m^2^q12h*3d) chemotherapy was administered. His head mass reappeared approximately 1 month after treatment with blinatumomab. MRD assessment revealed 91.5% primitive and immature monocytes, no immature lymphocytes, and no expression of CD19, indicating a lineage transformation. The child died of primary disease progression 2 months after treatment with blinatumomab.

**Case 7**: A boy aged 11 years and 10 months was presented to the hospital with cervical lymphadenopathy and an elevated leukocyte count. The patient was diagnosed with ALL (B, high-risk, MLL-AF17) according to the leukemia MICM classification. Then the patient received chemotherapy with the CCCG-ALL-2015 (12) regimen. The patient was unable to achieve molecular remission after multiple lines of chemotherapy. The patient had a complete relapse 9 months after diagnosis. Unfortunately, CR was not achieved at the end of the reinduction regimen (idarubicin, dexamethasone, Pegaspargase, vincristine). After informed consent, the patient was enrolled in a clinical trial (NCT04888468). The FC regimen was used to remove lymphocyte chemotherapy before infusion, and then the patient received anti-CD19 CAR T cells with 0.6 × 10^6^/kg. The expansion of anti-CD19 CAR T cells in the peripheral blood is shown in Figure 3D. Grade 2 CRS (fever, hypotension, and hypoxia) occurred on the seventh day after infusion. These symptoms were relieved with glucocorticoids, rehydration, and oxygen therapy. The patient developed several upper respiratory tract infections following CAR T-cell treatment. The patient achieved CR with MRD-negativity and maintained it for 3 months. Unfortunately, cervical lymphadenopathy reappeared on day 177 following CAR T cell infusion. Subsequently, the patient relapsed as shown by assessing FCM of 94.6% MRD (naive B cells partially expressed CD19). The family was lost to follow-up after discontinuing treatment.

## Discussion

CAR T-cell therapy refers to the genetic modification of patients or allogeneic T cells *in vitro*, followed by adoptive transfer back to the patient. CAR binding to tumor cell surface antigens activates CAR T cells and triggers the T-cell response of tumor cells expressing the antigen. Blinatumomab is an anti-CD3 × anti-CD19 fragment bispecific T-cell engager that links human CD3 + T cells with CD19 + leukemia cells, triggering a cytotoxic immune response and exerting antitumor effects (13). Both CAR T cells and blinatumomab use antibodies or antibody fragments to redirect T cells to specific tumor-associated antigens for targeted antitumor effects.

Immunotherapy has a higher response rate than traditional chemotherapy (5, 14–16). Compared to blinatumomab, CD19-targeted CAR T-cell therapy seems more effective (17), which may be related to the additional advantage of T-cell activation by CAR T-cell therapy. This study showed that the MRD-negative remission rate was as high as 100% in the three patients who received CAR T-cell therapy. Two of the other four children who received blinatumomab achieved complete molecular remission.

Immunotherapy was observed, revealing an excellent remission rate for relapsed/refractory special ALL types (such as Ph-positive and infantile leukemia). Zhang et al. (18) revealed that 14 patients with Ph-positive ALL were in CR after receiving anti-CD19 CAR T-cell treatment. Another study of Ph-positive ALL in adult patients resistant to tyrosine kinase inhibitors (19) revealed a CR rate after 2 weeks of blinatumomab treatment. A retrospective study from the United Kingdom and the Republic of Ireland (20) revealed that 11 infants with ALL were treated with one or two cycles of blinatumomab, and nine achieved MRD-negative CR. Moskop et al. (21) reported a 64% response rate to anti-CD19 CAR T-cell therapy in infants with B-ALL. In this study, the boy resistant to dasatinib Ph-positive ALL (case 5) was administered blinatumomab because of the previous leukoencephalopathy. Nevertheless, his treatment was discontinued because of ICANS. Case 6 was initially diagnosed with MPAL (B/M), and some immature lymphocytes remained after chemotherapy. After receiving blinatumomab treatment for 4 weeks, lineage transformation occurred, and the infant eventually died of the primary disease. Recently, some scholars (22, 23) explored blinatumomab combined with other antibodies to achieve complete molecular remission in relapsed/refractory ALL or MPAL. Dual-targeted antibody therapy may be an option for some patients with refractory and relapsed ALL or MPAL; however, many prospective studies still need to be clarified.

The use of CAR T-cell therapy has obvious advantages in children with extramedullary relapsed ALL. Leahy AB (24) revealed that anti-CD19 CAR T-cell therapy could treat CNS diseases and maintain durable remission without increasing the risk of severe neurotoxicity. Further, Talekar MK (25) confirmed that CAR T cells could migrate to extramedullary sites in patients with extramedullary relapse ALL and induce potent and durable responses. In this study, the patient in case 2 had a combined central relapse (CNS3) and bone marrow relapse. After undergoing triple intrathecal chemotherapy, the patient achieved CNS remission and received CAR T-cell therapy and HSCT treatment. Unfortunately, approximately 14 months after HSCT, the patient experienced a bone marrow relapse.

Adverse immunotherapy-related events mainly include CRS caused by antigen-specific T-cell activation, which results in the subsequent release of proinflammatory cytokines and ICANS (8). Blinatumomab is safer because CAR T-cell treatment is more prone to high-grade CRS and neurotoxicity (17). Approximately 15% of patients receiving blinatumomab treatment may develop grade 3 or higher ICANS, which may be relieved with discontinuation and dexamethasone treatment (26). The findings of our study revealed that three patients who received CAR T-cell infusion had grade 2 CRS without ICANS. Four children received blinatumomab infusion; two developed grade 1 CRS, and one with a history of leukoencephalopathy (case 5) developed grade 3 ICANS. The symptoms of the patient were relieved after drug discontinuation. Hypogammaglobulinemia due to B-cell dysplasia is a late adverse event of immunotherapy. These children have a significantly higher risk of infection due to secondary humoral immunodeficiency. A recent retrospective study (27) revealed that the rate of febrile infection in patients during peri-CAR T-cell treatment using NGS detection was as high as 77.45%, of which viral infection was the most common. In this study, three patients who received CAR T-cell treatment had hypogammaglobulinemia for up to approximately half a year, two patients had respiratory tract infections, and one experienced an intestinal infection during the study period. Four patients administered blinatumomab had no apparent infections. After immunotherapy, five children who underwent sequential HSCT were infected. The infection includes upper respiratory tract infection, pneumonia, and enteritis. The pathogens involved were viruses (CMV activation, EBV activation, HHV-5, HHV-6, adenovirus), Candida albicans, and Pneumocystis jirovecii. One patient who underwent anti-CD19 CAR T-cell therapy bridging HSCT recovered immune globulin 1 year after HSCT, and the other patient underwent donor anti-CD19/anti-CD22 CAR T-cell cocktail therapy again after HSCT and still had hypogammaglobulinemia 2 years after HSCT. More consideration should be given to the timing of immune reconstitution following immunotherapy, especially after bridging HSCT.

Immunotherapy has been shown to be highly effective in inducing marrow responses in patients with relapsed/refractory ALL; however, sustained remission is limited (28, 29), and some patients relapse. Schultz LM (30) revealed that the 1-year cumulative recurrence rate of anti-CD19 CAR T-cell therapy in children and adolescent patients with B-ALL was as high as 37% (CD19 positive was 21%, CD19 negative was 16%). CD19 negative recurrence and lower overall survival related. Blinatumomab appears to be a promising choice for consolidation therapy before allogeneic HSCT in children with relapsed or refractory B-ALL with residual MRD because it has a short half-life and no follow-up effect after discontinuation of administration (31). CAR T-cell therapy theoretically allows for long-term engraftment and provides a sustained source of tumor-reactive T cells to attack recurring tumors. Recurrence after CAR T cells is expected due to factors such as depletion of function and loss of target antigens. A study by Hay KA (32) revealed that patients with high lactate dehydrogenase levels, low platelet counts, and multiple lines of therapy before CAR T-cell therapy had poor EFS. Multiple studies have revealed that risk factors for relapse in CAR T-cell treatment include leukemia with *TP53* mutations and complex genetic profiles (>2 mutations), increased risk of lineage switch, pre-existing antigen-negative subclones (e.g., MLLr, Ph-positive B-ALL), and history of blinatumomab treatment (33–35). After an assessment, two of the three relapsed patients in this study showed CD19 negative relapse, and one showed CD19 antigen reduction. Two relapsed children showed MLLr, and one had a *TP53* mutation.

Controversy remains on whether consolidation therapy should be followed by CAR T-cell therapy. Some studies (36) showed no difference in EFS after CAR T-cell treatment remission between bridging HSCT and non-bridging HSCT groups. In contrast, others suggested that patients could benefit from HSCT following CAR T-cell treatment (37, 38). Recommendations and decisions on whether to consolidate CAR T cells with HSCT should be made following a comprehensive evaluation of patient factors (comorbidities, donor availability), disease (MRD status, B cell hypoplasia, previous HSCT), and CAR T-cell (costimulatory domain, potential persistence of CAR T cells) and other factors (39). In this study, we recommended HSCT after CAR T-cell treatment for case 7 with high-risk factors. Unfortunately, the family of the patient in case 7 discontinued the treatment after the second relapse due to a lack of a suitable donor. Case 1 was treated with anti-CD19/anti-CD22 CAR T-cell cocktail therapy in this study, followed by HSCT for approximately 6 months after infusion. The patient received donor anti-CD19/anti-CD22 CAR T cells due to PTLD. No evidence of high-grade CRS or ICANS, and aGVHD was grade I was observed. The patient is still alive with no adverse events. The safety and efficacy of anti-CD19/anti-CD22 CAR T cells and donor CAR T-cell infusions were verified.

Blinatumomab has higher controllability, fewer adverse effects, and better safety in children and infants from the time of approval by the U.S. Food and Drug Administration on March 29, 2018, for treating patients with B-cell precursor ALL in morphological remission with MRD (40). Blinatumomab may be considered an alternative to intensive chemotherapy for children with severe infections who cannot tolerate chemotherapy toxicity. Blinatumomab has been used as an effective bridging therapy before HSCT because of its ability to induce deeper molecular remission. CAR T-cell has a high effective remission rate in treating isolated and combined extramedullary relapsed ALL. However, the duration of remission is limited. Some patients with highly proliferative tumors may miss the best time for treatment due to the high production cost and long cycle. In addition, some patients have poor-quality CAR T cells due to defects in their T cells after receiving multiline therapy, which may affect the efficacy of the final CAR T-cell treatment. Findings from the current dual-targeted antigen CAR T-cell treatment, allogeneic CAR T-cell therapy combined with HSCT and universal CAR T-cell research findings may result in CAR T-cell breakthroughs (41).

## Conclusion

For pediatric ALL with rapid progression or high-risk relapsed factors, blinatumomab may be an ideal treatment to achieve deeper remission followed by bridging transplantation. CAR T-cell therapy may be better for children with extramedullary lesions. Considering multiple factors, the decision to bridge transplantation after CAR T-cell therapy needs to be made. CAR T-cell targeting CD19 and CD22 antigens and allogeneic CAR T-cell bridging HSCT may be new options.

## Data Availability

The original contributions presented in the study are included in the article/Supplementary Material, further inquiries can be directed to the corresponding author/s.
